# Old Dilemmas, New Commitments: Toward a 21st Century Strategy for Community Health Promotion

**Published:** 2007-06-15

**Authors:** Janet L Collins, H. Wayne Giles, Amy Holmes-Chavez

**Affiliations:** Centers for Disease Control and Prevention, National Center for Chronic Disease Prevention and Health Promotion, Office of the Director; Centers for Disease Control and Prevention, National Center for Chronic Disease Prevention and Health Promotion, Atlanta, Ga; Centers for Disease Control and Prevention, National Center for Chronic Disease Prevention and Health Promotion, Atlanta, Ga

The recommendations of the National Expert Panel on Community Health Promotion (expert panel) (1,2), convened in March 2006 in Atlanta, could not have come at a more critical time for the Centers for Disease Control and Prevention's (CDC's) National Center for Chronic Disease Prevention and Health Promotion (NCCDPHP). During the past several decades, U.S. society has undergone profound changes, and these changes affect how NCCDPHP must operate: rapid advances in technology make Americans increasingly sedentary; the ever-increasing pace of life creates stress; and the beginning of a demographic shift toward an older population increases demands on limited resources. In addition, we must continue to address persistent and troubling disparities in health, health care, and related social factors such as poverty, violence, low educational attainment, and family stress. Each of these factors could escalate a growing national crisis in chronic disease with far-reaching effects not only on individuals and families but also on the U.S. economy. In light of these challenges, CDC welcomes the recommendations of the expert panel (1). They will help CDC to design a more efficient and strategic approach to promoting health and preventing chronic disease.

## Measuring Community Health Risks

In the 1980s, state health departments and CDC collaborated to produce state data that track behavioral risks for various health conditions by implementing the Behavioral Risk Factor Surveillance System (BRFSS). BRFSS and its companion, the Youth Risk Behavior Surveillance System (YRBSS), drew the attention of state and federal policy makers to behaviors that are risk factors for chronic disease. Policy makers responded by passing legislation and launching public health initiatives on issues ranging from reducing tobacco use to increasing seat belt use and from mandating insurance coverage for mammography to combating the obesity epidemic. However, as we deepen our understanding of how societal issues, public health interventions, and public policy decisions affect trends in health status, we must establish complementary data systems that assess and monitor these factors and the extent of their effect on population health.

## Linking Research and Practice

The expert panel recommends an increased commitment to community participation in public health research. Experience with the Prevention Research Centers and programs such as REACH (Racial and Ethnic Approaches to Community Health) and Steps to a HealthierUS demonstrate that community engagement in the design and evaluation of prevention activities pays off by producing innovative approaches to improving people's health and reducing seemingly intractable health disparities. The challenge of the coming years will be to link lessons learned from involving communities in research with the daily work of programs throughout the field of public health.

## Moving From Recognition to Action

The expert panel confirmed that daily living conditions influence the ability to make healthy behavioral choices, which determines health status. However, it is not enough to document the impact of social factors on population health; action is necessary to promote protective factors and minimize the influence of social and environmental barriers to health. For example, public health programs that provide people with information about risky behaviors must be complemented by programs to design supportive social and physical environments (e.g., school systems, workplaces, health care systems). Although the ability of certain environmental and social changes to improve population health has long been recognized, the skills needed to bring about these changes are only now being learned and institutionalized. The expert panel calls for a renewed commitment to learn, teach, and establish institutional skills in preventing society-related illnesses.

## Bringing About Social Change  

To achieve the societal and environmental changes that will improve population health requires an understanding of how to engage with organizations outside the purview of public health and influence their decisions, become an active participant in policy-making processes in a wide range of organizations and at all levels of government, and enable communities to implement strategies that will improve factors that contribute to good health. For many public health professionals, this is new territory. CDC's challenge will be to increase the effects of public health programs by seeking expanded skills and expertise in the theory and practice of social change and working to integrate that expertise into public health practice.

## New Solutions to Old Dilemmas 

The expert panel discussed some tensions that we have lived with for years, including the tension between a community's need for a comprehensive array of health interventions and Congress's penchant for categorical funding. The expert panel also reminded us of the need to address the conflict between the wellness of the whole person (i.e., focus on physical, mental, and emotional health) and the limited models and resources available to meet these needs. Programs such as Steps to a HealthierUS, REACH, and the Preventive Health and Health Services Block Grant offer important and exciting opportunities to begin to fill these knowledge gaps.

## Public Health for the 21st Century

We are on the cusp of a transformation in public health from a field that focuses on the individual to one that focuses on the societal issues tied to health and illness. The recommendations of the expert panel will help to advance this new vision of public health action. We extend our sincere thanks to the members of the panel for their contributions and commitment.
